# Identification of *FASN* Gene Polymorphisms, Expression and Their Relationship with Body Size Traits in Guizhou White Goat (*Capra hircus*) with Different Genders

**DOI:** 10.3390/genes15060656

**Published:** 2024-05-22

**Authors:** Qingming An, Lingli Zeng, Wenying Wang, Jiangyu Yang, Jinzhu Meng, Yuanyuan Zhao, Xingchao Song

**Affiliations:** Guizhou Provincial Key Laboratory for Biodiversity Conservation and Utilization in the Fanjing Mountain Region, College of Agriculture and Forestry Engineering, Tongren University, Tongren 554300, China; zll1112222024@163.com (L.Z.); w19985578865@163.com (W.W.); 84840293@163.com (J.Y.); mjz122821@126.com (J.M.); yjy13251252247@163.com (Y.Z.); songxingchao_888@126.com (X.S.)

**Keywords:** *FASN* gene, nucleotide variation, body size traits, goat

## Abstract

To investigate the nucleotide variation sites (SNPs) and expression differences of the fatty acid synthase gene (*FASN*) in Guizhou white goats, the relationship between the variation and body size traits was investigated. In this study, DNA was extracted from the blood of 100 samples of white goats from different regions in Guizhou province, China, and the variation sites were screened using pooled sequencing by mixing DNA samples, and 242 blood samples with body size traits were used for association analysis. The allele frequency, genotype frequency, homozygosity, heterozygosity and effective gene number were calculated by using PopGene 32.0 software, the population polymorphism information content was calculated by using PIC software (Version 0.6), and the state of genetic balance of the genes was analyzed by using the chi-square test. The mRNA of *FASN* gene expression levels in male and female goats were investigated by using real-time fluorescence quantitative PCR (RT-qPCR). The general linear mixed model of MINTAB software (Version 16.0) was used to analyze the association between *FASN* gene nucleotide mutation sites and body size traits. The results showed that there was one nucleotide mutation site g.141 C/T in the target fragment of *FASN* gene amplification, and revealed two alleles, C and T, and three genotypes CC, CT and TT. The genotype frequencies for CC, CT and TT were 0.4308, 0.4205 and 0.1487, respectively. The allele frequencies for C and T were 0.6410 and 0.3590, respectively. The genetic homozygosity (Ho) was higher than the heterozygosity (He). The χ^2^ test showed that the mutation site was in the Hardy–Weinberg equilibrium state (*p* > 0.05). The RT-qPCR results showed that the *FASN* gene had different expression levels in the longissimus dorsi muscle of male and female goats, and its expression was significantly higher in male goats than in female goats. The association analysis results showed that the mutation of the *FASN* gene had different effects on body size traits of male and female goats, and the presence of the populations of the T allele and the TT genotype recorded higher body size traits (body weight, heart girth and wither height) in female populations. Therefore, the site of the *FASN* gene can be used as a candidate marker for the early selection of growth traits in Guizhou white goats.

## 1. Introduction

Fatty acid synthase (FASN) is an enzymatic structure that catalyzes the synthesis of fatty acids, and can utilize acetyl-CoA and malonyl-CoA to synthesize long-chain fatty acids from scratch in the presence of NADPH. FASN is a key enzyme in the synthesis process, and most of the fatty acids required for animal fat deposition need to be synthesized de novo [1,2]. The *FASN* gene is located on chromosome 19 of the goat, with a full-length sequence of 18,795 bp, encoding a total of 2514 amino acid residues (RefSeq accession GCF_001704415.1). Studies have found that the protein involved in the expression of the *FASN* gene is a water-soluble and unstable protein, lacking signal peptides and transmembrane regions, with 20 amino acids that can serve as protein kinase phosphorylation sites [3,4]. Many researchers have conducted studies on the important regulatory role of the *FASN* gene in fatty acid synthesis in animal production. It was found that there is a correlation between the polymorphism of the *FASN* gene and fat tissue deposition in pigs [5]. Moreover, some studies have found that the expression correlation analysis of *FASN* gene in 10 tissues including sheep tail fat, heart, kidney, liver, etc., showed that the *FASN* gene is expressed in all 10 tissues, and the expression quantity in tail fat is significantly higher than in the other 9 tissues [6,7,8]. In the cattle population, it has also been found that the *FASN* gene may have a significant impact on cattle carcass traits, with research indicating that there is a certain correlation between *FASN* gene variation and carcass traits, body size traits and intramuscular fat deposition in Qinchuan cattle [9,10].

Therefore, the *FASN* gene is an important candidate gene that affects the production performance of livestock animals. However, there are few reports on the study of the *FASN* gene in goats, and there is currently no report on the analysis of *FASN* gene variation and its relationship with growth traits in goats. Guizhou white goats, a famous local goat breed in China, play an important role in the development of the local livestock industry. Thus, this study aims to conduct research on the *FASN* gene in Guizhou white goats, analyze the nucleotide variations and tissue expression differences of the *FASN* gene in the Guizhou white goat population, and explore its correlation with the growth traits in Guizhou white goats with different genders in order to identify molecular genetic marker points that can help us to select goats with better growth traits in the early stages.

## 2. Materials and Methods

### 2.1. Animals and Data Collection

In order to explore the genetic variation in the goat *FASN* gene, 342 blood samples were collected and brought back to the laboratory for storage at −20 °C. Among these, 100 blood samples were collected to be used for nucleotide mutation detection from 7 different farms; another 242 blood samples were collected from 2 farms, and on each farm, the goats were randomly selected, with the age generally between 2 and 2.5 years old. Basic body size trait data of Guizhou white goats (body weight, heart girth, wither height, body length and circumference of cannon bone) for the corresponding individuals were also recorded. DNA extraction was performed using the DNA Extraction Kit (TIANGEN Biotech, Beijing, China). The DNA from 100 samples of the 7 different farms was diluted to the same concentration with TE and 2 μL was taken for mixing to prepare mixed DNA for subsequent pooled sequencing. Another 242 DNA samples were used for the analysis of growth trait association. All DNA samples were stored at −20 °C prior to use. In addition, 3 male and 3 female Guizhou white goats’ longissimus dorsi tissues were collected, respectively. Before slaughter, we measured the live weight, and after slaughter, we measured the carcass weight, meat weight, calculated yield and net meat rate. The samples were immediately preserved in liquid nitrogen after collection until RNA extraction. The total RNA was isolated from longissimus dorsi tissue using the RNAsimple Total RNA Kit (TIANGEN Biotech, Beijing, China) following the manufacturer’s protocols. Aliquots (200 ng) of total RNA were used for cDNA synthesis using TransScript One-Step gDNA removal and cDNA Synthesis SuperMix (TransGen Biotech, Beijing, China) following the manufacturer’s protocols.

### 2.2. Primer Design and PCR Amplification

Using the *FASN* gene sequence information for goats from the NC_030826.1 assembly in the NCBI database as a reference, the appropriate primer fragment length was selected, containing part of the 21st exon and part of the 21st intron, and primer 5.0 software (Version 5.0, Premier Biosoft International, Palo Alto, CA, USA) was used to design the PCR primer, which was synthesized by the Sangon Biotech Service Company (Chengdu, China), to amplify the fragment of the *FASN* gene. The primer information is shown in Table 1 (the primer P1, and after screening for polymorphic sites, a new primer P2 was designed for precise detection, containing nucleotide mutation sites). The primers were dissolved in ultrapure water and stored at −20 °C.

The PCR amplification was conducted using 2 × Taq PCR StarMix, with a reaction volume of 40 μL, which contained 2 μL of DNA template, 22 μL of 2 × Taq PCR StarMix, 2 μL of upstream primers, 2 μL of downstream primers and 12 μL of ddH_2_O. The PCR reaction process includes pre-denaturation at 95 °C for 5 min, denaturation at 95 °C for 30 s, annealing at 57 °C for 30 s and extension at 72 °C for 30 s for 40 cycles, final extension at 72 °C for 10 min for 1 cycle, and preservation at 10 °C. The amplified products of the PCR were analyzed by using 1.5% agarose gel electrophoresis for 15 min. 

RT-qPCR detects the relative expression level of the *FASN* gene using a fluorescent quantitative PCR detection kit produced by TransGen Biotech Co., Ltd. (Beijing). The amplification system is 20 μL: cDNA template (1 μg/L) at 1 μL, 2 × PCR super mix at 10 μL, upstream and downstream primers (10 μmol/L) at 0.5 μL, respectively, and ddH_2_O at 8 μL. The RT-qPCR reaction process includes 94 °C denaturation for 1 min for one cycle, followed by 94 °C for 10 s, 58 °C for 30 s and 72 °C for 10 s for 38 cycles. Three technical repetitions were run for each sample and the results were normalized to *GAPDH*. The primers’ information of the PCR and RT-qPCR of the *FASN* gene is given in Table 1.

### 2.3. Measurements and Statistical Analysis

The mutation sites of the *FASN* gene were analyzed by the MegAlign software with DNASTAR lasergene (Versionn7.1.0, DNASTAR, Inc., Madison, Wisconsin, USA). Genetic diversity parameters (including genotype frequency, allele genotype frequency, observed homozygosity (Ho), expected heterozygosity (He) and Hardy–Weinberg test (χ^2^ test)) were calculated by using PopGene 32.0 software (Molecular Biology and Biotechnology Centre, University of Alberta, Edmonton, AB, Canada). The polymorphic information content (PIC value) was calculated by using PIC software (PIC_CALC, Version 0.6, Yellow Sea Fisheries Research Institute (YSFRI), Chinese Academy of Fishery Sciences, Wuhan, China). General linear mixed-effect models (GLMMs) of MINITAB (Version 16, Minitab Inc., State College, PA, USA) were used to evaluate the effect of the presence/absence of the allele on the body size traits. For genotypes with a frequency greater than 5%, a series of GLMMs was used to evaluate the effect of the allele on the body size traits. The model used in the analysis is as follows:

Y_ijmn_ = μ + C_i_ + F_m_ + X_n_ + e_imn_

Among them, Y_ijmn_ represents the corresponding productive traits, μ represents the population mean, C_i_ represents the gender effect, F_m_ represents the genotype and allele effect, X_n_ represents the interaction effect between factors and e_imn_ represents the random error. All results are expressed as “mean ± standard error”.

Differential expression analysis was performed as follows: ^ΔΔ^Ct = (Ct ^*FASN* gene^ − Ct ^*GAPDH*^) for the experimental group and (Ct *^FASN^* ^gene^ − Ct ^*GAPDH*^) for the control group. The raw data were first processed using Excel 2019 software, and then, the 2^−ΔΔCT^ method was used to calculate the relative gene expression level. One-way ANOVA was used to analyze the results for significance, with a significant difference established when *p* < 0.05.

## 3. Results

### 3.1. Analysis of the Polymorphism of the FASN Gene

**Figure 1:**
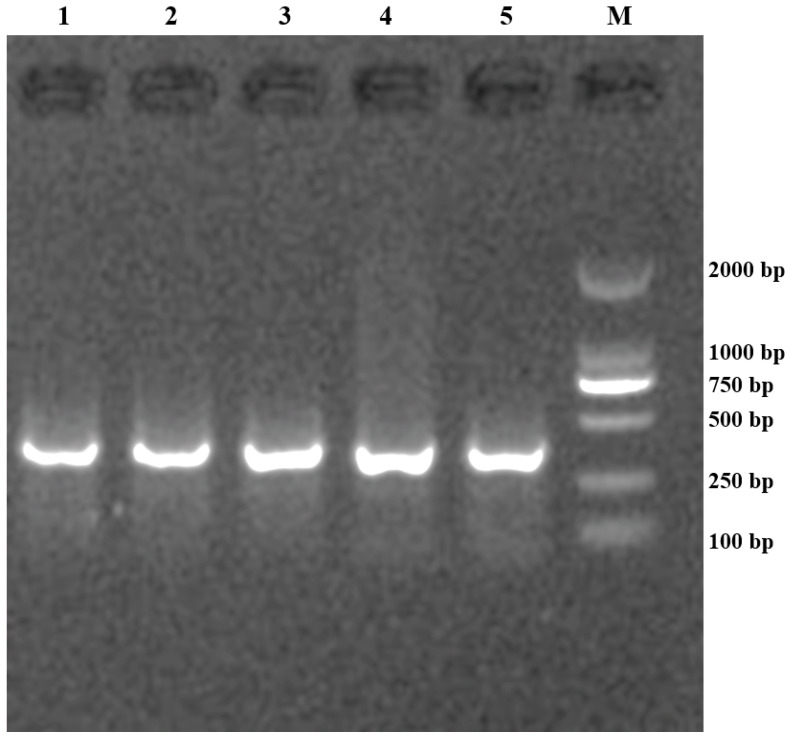
Agarose electrophoresis of PCR products of goat *FASN* gene P1 target fragment.

**Figure 2:**
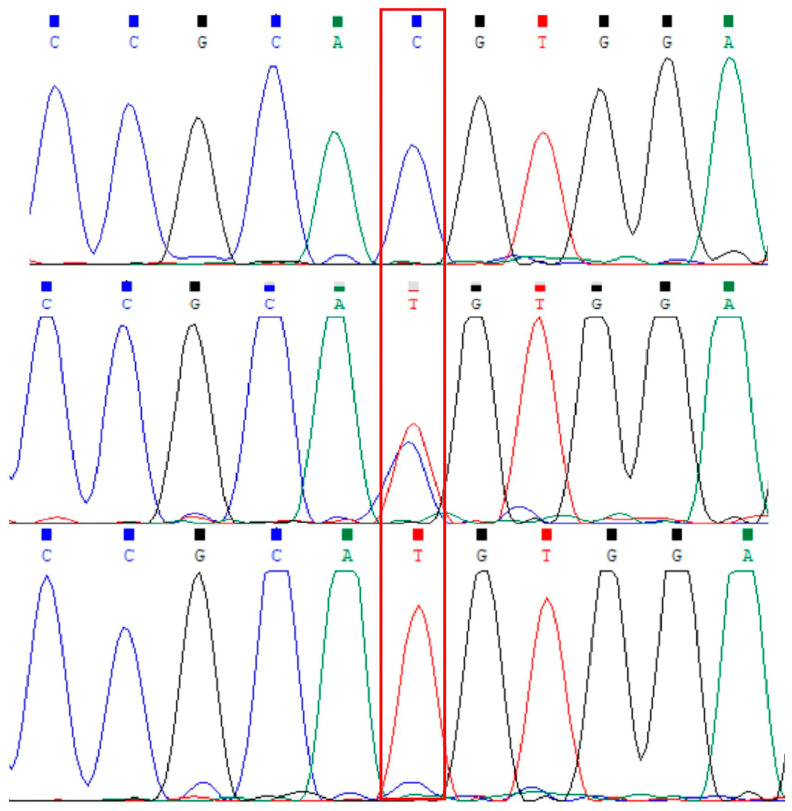
The sequencing maps of the novel SNP in the goat *FASN* gene. A schematic representation of the *FASN* gene sequence with the novel position identified, the peaks in the red box represent the nucleotide variation sites.

We used 1.5% agarose to detect the PCR products. The amplified bands were clear and could be used for subsequent tests (Figure 1). A g.141C/T mutation or natural cleavage site was found in the region of exon 21 of the *FASN* gene by comparing the sequence of the P1 target fragment with the reference sequence (10614th position of NC_030826.1) and then through the verification of the target gene fragment amplified by the P2 primer (Figure 2). It is notable that the g.141C/T mutation is a synonymous mutation. 

The 100 samples from 7 different farms were used to detect nucleotide variation sites through a pooled approach, and hence, no population genetic information was analyzed. In the 242 individuals of Guizhou white goat, there were three genotypes that were sequenced. These were CC (43.59%), TT (14.36%) and CT (42.05%), respectively. Meanwhile, the g.141C/T mutation signified that there were two different alleles C and T, and the frequencies were 64.6% and 35.4%, respectively (Table 2). 

As shown in Table 2, the calculated polymorphic information content (PIC) value is 0.35 (PIC > 0.5 indicates high polymorphism, 0.25 < PIC < 0.5 represents moderate polymorphism, and PIC < 0.25 reflects low polymorphism), indicating that the PIC of this variant site is moderately polymorphic. Moreover, the genetic homozygosity (Ho) is greater than the genetic heterozygosity (He), and Ne is 0.4573. The chi-square test (χ^2^) indicates that the locus frequency is in the state of Hardy–Weinberg disequilibrium rather than Hardy–Weinberg equilibrium (*p* > 0.05).

### 3.2. Analysis of the Differential Expression Level of the FASN Gene in Male and Female Goats

**Figure 3:**
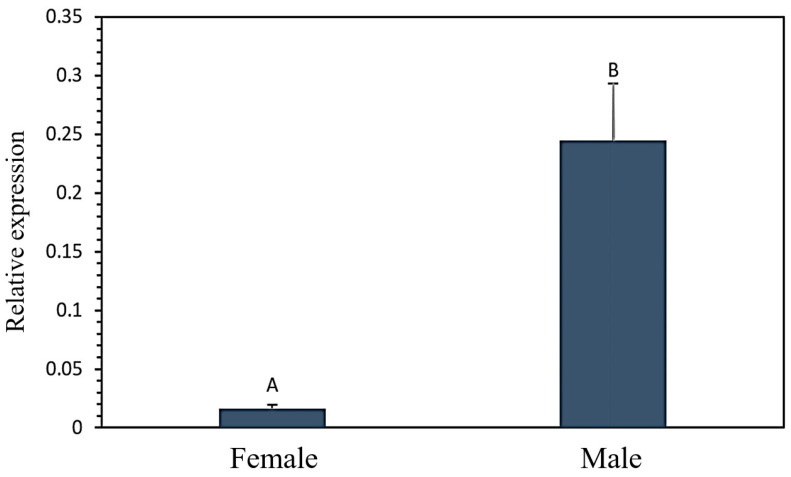
The mRNA relative expression level of *FASN* in longissimus dorsi tissue of Guizhou white goats. Different letters above the error bars indicate significant differences at the *p* < 0.05 level.

The mRNA expression level of the *FASN* gene was detected in longissimus dorsi tissue in male and female goats by using RT-qPCR. The results showed that the expression level has a gender-specific feature, with significantly lower expression in females than in male goats (Figure 3).

### 3.3. Effect of Variation in FASN on Body Size Traits

In the allele (presence/absence) model, the presence of C was associated with decreased body weight (*p* < 0.01), heart girth (*p* < 0.01) and wither height (*p* = 0.04). The presence of T was associated with increased body weight (*p* < 0.01) and heart girth (*p* = 0.05) (Table 3). The genotype was found to have an effect on body weight (*p* < 0.01) and heart girth (*p* = 0.01). The TT genotype resulted in a higher body weight and heart girth compared to those with the genotypes CC and CT (Table 4). No associations were found between the other *FASN* alleles/genotypes and body length and circumference of the cannon bone.

The results showed that the effect of variation in *FASN* gene on body size traits has a gender-specific feature. In the allele (presence/absence) model, the presence of C was associated with decreased body weight (*p* < 0.01), heart girth (*p* = 0.02), wither height (*p* < 0.01) and body length (*p* = 0.03) in female goats. The presence of T was associated with increased body weight (*p* < 0.01) in females (Table 5). The TT genotype resulted in a higher body weight (*p* < 0.01), heart girth (*p* = 0.05), wither height (*p* < 0.01) and body length (*p* = 0.04) compared to those with the genotypes CC and CT in female goats (Table 6). But no associations were found between the *FASN* alleles/genotypes and body size traits.

## 4. Discussion

The Guizhou white goat is a prized local breed with significant economic value, which is currently the largest local goat breed in Guizhou province, China. However, the breed faces challenges that impede its industrial advancement, including a protracted growth rate, diminished physical robustness, and a suboptimal feed utilization rate, all of which severely limit the development of the Guizhou white goat industry. The *FASN* gene, a pivotal player in the lipid metabolic pathway, encodes the enzyme fatty acid synthase. This enzyme catalyzes the de novo fatty acid synthesis, initiating from acetyl coenzyme A and malonyl-CoA, which is integral to the bioenergetic and structural requirements of the organism. The FASN-mediated biosynthetic pathway is not only fundamental to the assembly of cell membranes but also underpins energy storage and signal transduction mechanisms [1,3,11]. Throughout the ontogenetic trajectory of animals, the activity of the *FASN* gene is a determining factor in adipose tissue formation and energy deposition, directly influencing the growth trajectory, feed conversion efficiency and overall health of the animals [12,13,14]. Given the cardinal role of the *FASN* gene in regulating metabolic balance and growth, an investigation into the effects of its genetic variations on the growth performance of Guizhou white goats is both pertinent and timely; this could contribute to the economic potential and sustainability of the Guizhou white goat industry.

This study focuses on different groups of Guizhou white goats, identifying a nucleotide variation in exon 21 of the *FASN* gene, which is characterized as a synonymous mutation. Although the mutation site does not alter the amino acid sequence of the resultant protein, it may still have implications for gene expression and regulation, a phenomenon that warrants further exploration. The PIC at this specific locus has been calculated and is classified as moderately polymorphic for the population (0.5 > PIC > 0.25). This suggests considerable genetic diversity that could be harnessed through targeted breeding programs to enhance desirable traits. Despite intentional selection pressures exerted by breeders throughout the breed’s evolutionary history, the population remains in Hardy–Weinberg equilibrium. This equilibrium indicates a potential for future selective breeding initiatives aimed at improving production traits, such as growth rate, meat quality and carcass traits.

In this study, an analysis of *FASN* gene expression levels in the longissimus dorsi tissue of goats across different sexes revealed a marked disparity, with rams exhibiting significantly higher expression levels than ewes. This discovery holds significant implications for understanding the genetic underpinnings of growth differences between sexes in goats. Given that the *FASN* gene encodes a critical enzyme involved in the regulation of fatty acid synthesis rates, its elevated expression levels are typically associated with accelerated growth of muscular and adipose tissues. Empirical breeding observations corroborate this finding, as rams are known to surpass ewes in terms of growth velocity and meat production during their rapid growth phase. This sexual dimorphism in *FASN* gene expression aligns with the biological traits and provides indirect evidence that the *FASN* gene is an important candidate gene in the regulation of caprine growth. This correlation between *FASN* expression and growth characteristics is further supported in this study. Li et al. [15] have also reported gender-specific expression patterns of *FASN* in healthy adults which were positively correlated with increased serum triglyceride levels. Similarly, some studies have found that the *FASN* gene has different expression levels in pigs, Yanbian Yellow cattle and Gannan Yak, which reveals that the *FASN* gene is highly expressed in adipose tissue [16,17,18]. These findings indicate variable expression levels among breeds and tissues, which could contribute to the present result of its higher expression level in perirenal adipose tissue.

Extensive research has consistently revealed significant correlations between polymorphisms within the *FASN* gene and the growth performance parameters of domesticated animals. Aimaamory et al. [19] have provided compelling evidence that the A/G mutational site within the *FASN* gene exerts a significant influence on body weight and weight gain between birth and weaning of Awassi Sheep. This underscores the potential role of the *FASN* gene in modulating growth traits in Awassi Sheep. In the porcine domain, several studies have identified a significant correlation between the mutation locus on the porcine *FASN* gene and back fat fatty acid composition, feed conversion, meat color traits and back fat thickness [5,20,21]. These associations suggest that the *FASN* gene may be a critical determinant of meat quality attributes. In cows, *FASN* gene mutations have been correlated with alterations in milk fat content and the proportional composition of primary milk constituents [17,22]. In Qinchuan cattle, it has also been found that FASN gene mutations are significantly associated with growth, carcass traits, body size characteristics and intramuscular fat [9,10]. Therefore, these findings collectively substantiate the *FASN* gene’s role as a pivotal candidate gene impacting the growth traits of livestock. Building upon this foundation, in this study, the g.141C/T mutation within the *FASN* gene was identified to have a significant association with body weight and heart girth in Guizhou white goats. This association was found to be gender-specific, with the female population exhibiting a pronounced correlation between variations at this site and body weight, wither height, body length and heart girth, which could have important implications for sex-specific breeding strategies.

## 5. Conclusions

In conclusion, the work presented in this study confirms a single mutation site, g.141C/T, in exon 21 of the *FASN* gene. This mutation was found to have a significant impact on body weight, wither height, body length and heart girth in Guizhou white goats. Additionally, the association and expression levels of the *FASN* gene in longissimus dorsi tissues were found to be gender-specific. Therefore, further research into the role of the *FASN* gene in determining growth traits seems to be warranted.

## Figures and Tables

**Table 1:** Primers information used for PCR and RT-qPCR.

Primer	Primer Sequences (5′ → 3′)	Tm (°C)	Product Length (bp)
*FASN*-P1	F:CTCTGGAAGGACAACTGGGT	57 °C	638 bp
	R:AAGAGGGGCGAAATCTGTCA		
*FASN*-P2	F:CCGACGTGGTAATGAACAGG	57 °C	361 bp
	R:CCCCTCAGAAGACATCAGCT		
*FASN*-β (RT-qPCR)	F:CTTGACACGGCTCAACTCG	58 °C	118 bp
	R:ATAGGTGGGGATGCTGAGC		
*GAPDH* (RT-qPCR)	F: GGCCTCCAAGGAGTAAGGTC	58 °C	124 bp
	R: CGGGAGATTCTCAGTGTGGT		

**Table 2:** Population genetic analysis of *FASN* gene in goats.

Genotype	Genotype Frequency	Allele	Allele Frequency	Ho	He	Ne	χ^2^	PIC
CC	43.08%	C	64.1%	0.5415	0.4585	0.4573	1.3437 (*p* = 0.25)	0.35
TT	14.87%	T	35.9%					
CT	42.05%							

**Table 3:** Association of *FASN* alleles with body size traits in goats.

Body Size Trait	Allele	Present	Absent	*p*-Value
Body weight (kg)	C	25.48 ± 0.73	32.71 ± 1.44	**<0.01**
	T	28.30 ± 0.87	24.82 ± 1.04	**<0.01**
Heart girth (cm)	C	69.19 ± 0.78	73.95 ± 1.48	**<0.01**
	T	71.33 ± 0.91	68.56 ± 1.07	**0.05**
Wither height (cm)	C	57.23 ± 0.70	60.12 ± 1.24	**0.04**
	T	57.81 ± 0.78	58.10 ± 1.04	0.82
Body length (cm)	C	51.14 ± 1.09	54.08 ± 2.04	0.19
	T	51.45 ± 1.23	52.21 ± 1.60	0.70
Circumference of cannon bone (cm)	C	7.55 ± 0.18	7.90 ± 0.35	0.37
	T	7.69 ± 0.20	7.51 ± 0.25	0.57

For each body size trait, *p*-values in bold are significant.

**Table 4:** Association of *FASN* genotypes with body size traits in goat.

Body Size Traits	Genotype	Mean ± Standard Error	*p*-Value
Body weight (kg)	CC	24.79 ± 1.0 ^a^	<0.01
	CT	26.18 ± 1.01 ^a^	
	TT	32.72 ± 1.44 ^b^	
Heart girth (cm)	CC	68.54 ± 1.06 ^a^	0.01
	CT	69.89 ± 1.11 ^ab^	
	TT	73.97 ± 1.48 ^b^	
Wither height (cm)	CC	58.09 ± 1.01	0.07
	CT	56.48 ± 0.95	
	TT	60.11 ± 1.24	
Body length (cm)	CC	52.34 ± 1.58	0.25
	CT	50.13 ± 1.45	
	TT	54.16 ± 2.04	
Circumference of cannon bone (cm)	CC	7.51 ± 0.25	0.65
	CT	7.59 ± 0.25	
	TT	7.90 ± 0.36	

In the same column, the values with different letter superscripts have a significant difference (*p* < 0.05).

**Table 5:** Association of *FASN* alleles with body size traits in male and female goats.

Body Size Trait	Allele	Male			Female		
		Present	Absent	*p*-Value	Present	Absent	*p*-Value
Body weight (kg)	C	30.41 ± 1.59	30.19 ± 2.11	0.93	28.42 ± 0.58	33.16 ± 1.24	**<0.01**
	T	30.47 ± 1.46	30.08 ± 1.80	0.84	30.55 ± 0.68	27.29 ± 0.86	**<0.01**
Heart girth (cm)	C	73.34 ± 2.89	71.35 ± 3.56	0.65	71.94 ± 0.63	75.20 ± 1.28	**0.02**
	T	73.20 ± 2.57	70.99 ± 3.59	0.57	75.27 ± 0.70	71.18 ± 0.96	0.08
Wither height (cm)	C	61.52 ± 2.10	57.96 ± 2.58	0.27	56.90 ± 0.64	61.51 ± 1.18	**<0.01**
	T	58.98 ± 1.84	63.12 ± 2.57	0.15	58.01 ± 0.75	57.67 ± 1.07	0.79
Body length (cm)	C	54.98 ± 1.70	53.81 ± 2.09	0.66	50.25 ± 1.27	58.50 ± 3.12	**0.03**
	T	54.01 ± 1.50	55.82 ± 2.10	0.43	50.90 ± 1.71	52.75 ± 2.70	0.57
Circumference of cannon bone (cm)	C	8.17 ± 0.29	7.96 ± 0.36	0.63	7.32 ± 0.18	8.05 ± 0.43	0.15
	T	8.11 ± 0.26	8.04 ± 0.36	0.87	7.41 ± 0.21	7.45 ± 0.34	0.92

For each body size trait, *p*-values in bold are significant.

**Table 6:** Association of *FASN* genotypes with body size traits in male and female goats.

Body Size Trait	Genotype	Male		Female	
		Mean ± Standard Error	*p*-Value	Mean ± Standard Error	*p*-Value
Body weight (kg)	CC	30.16 ± 1.88	0.97	27.29 ± 0.84 ^a^	<0.01
	CT	30.71 ± 1.98		29.45 ± 0.80 ^a^	
	TT	30.19 ± 2.13		33.16 ± 1.23 ^b^	
Heart girth (cm)	CC	71.45 ± 3.66	0.63	71.23 ± 0.95 ^a^	0.05
	CT	75.08 ± 3.55		72.48 ± 0.82 ^ab^	
	TT	71.29 ± 3.57		75.17 ± 1.28 ^b^	
Wither height (cm)	CC	63.25 ± 2.64	0.31	57.70 ± 0.97 ^a^	<0.01
	CT	59.94 ± 2.56		56.34 ± 0.82 ^a^	
	TT	58.01 ± 2.58		61.52 ± 1.18 ^b^	
Body length (cm)	CC	55.86 ± 2.16	0.73	52.75 ± 2.10 ^a^	0.04
	CT	54.18 ± 2.10		49.00 ± 1.49 ^ab^	
	TT	53.84 ± 2.11		58.50 ± 2.97 ^b^	
Circumference of cannon bone (cm)	CC	8.08 ± 0.37	0.82	7.45 ± 0.32	0.32
	CT	8.26 ± 0.36		7.25 ± 0.22	
	TT	7.95 ± 0.36		8.05 ± 0.45	

In the same column, the values with different letter superscripts have a significant difference (*p* < 0.05).

## Data Availability

The authors affirm that all of the data necessary for confirming the conclusions of this article are present within the article, figures and tables.
